# ASO Author Reflections: Does Ki67 Gene Expression Capture Immune Infiltration and Predict Chemotherapy Response in ER+/HER2− Breast Cancer?

**DOI:** 10.1245/s10434-026-19720-z

**Published:** 2026-04-27

**Authors:** Kohei Chida, Rongrong Wu, Kei Kawashima, Abigail Grapes, Li Yan, Itaru Endo, Takashi Ishikawa, Kenichi Hakamada, Kazuaki Takabe

**Affiliations:** 1 Department of Surgical Oncology, Roswell Park Comprehensive Cancer Center, Buffalo, NY USA; 2 Department of Gastroenterological Surgery, Hirosaki University Graduate School of Medicine, Hirosaki, Japan; 3 Department of Breast Surgery and Oncology, Tokyo Medical University, Tokyo, Japan; 4 Department of Gastroenterological Surgery, Yokohama City University Graduate School of Medicine, Yokohama, Japan; 5 Department of Surgery, University at Buffalo Jacobs School of Medicine and Biomedical Sciences, The State University of New York, Buffalo, NY USA; 6 Department of Biostatistics and Bioinformatics, Roswell Park Comprehensive Cancer Center, Buffalo, NY USA; 7 Department of Immunology, Roswell Park Comprehensive Cancer Center, Buffalo, NY USA; 8 Division of Digestive and General Surgery, Niigata University Graduate School of Medical and Dental Sciences, Niigata, Japan; 9 Department of Breast Surgery, Fukushima Medical University School of Medicine, Fukushima, Japan; 10 Department of Breast Surgery, Roswell Park Comprehensive Cancer Center, Buffalo, NY USA

## Past

Ki67 has long been used as a clinical marker of cellular proliferation, including in estrogen receptor-positive (ER+)/human epidermal growth factor receptor 2-negative (HER2−) breast cancer^1^. Ki67 assessed by immunohistochemistry has been associated with both increased response to neoadjuvant chemotherapy and worse long-term survival outcomes, reflecting its dual prognostic and predictive roles. However, the clinical utility of Ki67 assessed by immunohistochemistry has been somewhat limited by variability in measurement and interpretation, with no universally accepted cutoffs^2^. In contrast, transcriptomic assessment of the Ki67 gene (*MKI67*) provides a more objective and reproducible measure of proliferative activity^3^, but prior studies in ER+/HER2− breast cancer have been limited by small cohorts and lack of validation. Therefore, we sought to elucidate the clinical relevance of Ki67 at the transcriptomic level using large-scale, multi-cohort analyses^4^.

## Present

*MKI67* expression was validated across multiple large independent cohorts as a robust marker of cancer cell proliferation, demonstrating strong associations with higher histologic grade and enrichment of cell cycle-related pathways. Tumors with high *MKI67* expression also exhibited increased genomic instability, including higher mutation burden, homologous recombination deficiency, and greater intratumoral heterogeneity. Notably, *MKI67*-high tumors exhibited greater immune cell infiltration, suggesting a relationship between proliferative activity and the tumor immune microenvironment. Although survival differences were not consistently significant, *MKI67*-high tumors demonstrated higher rates of pathological complete response to neoadjuvant chemotherapy across multiple cohorts. Collectively, these findings indicate that Ki67 gene expression identifies a link between cellular proliferation, immune infiltration, and treatment response in ER+/HER2− breast cancer.

## Future

Ki67 gene expression may serve as a quantitative biomarker that reflects proliferative, genomic, and immunologic features to inform treatment response in ER+/HER2− breast cancer. However, prospective validation studies are required to establish clinically actionable thresholds and confirm its utility in guiding therapeutic decision-making. Further mechanistic studies are also needed to delineate the relative contributions of tumor-intrinsic proliferation and immune-mediated effects on treatment response and survival outcomes. Additionally, standardized approaches for transcriptomic normalization and integration with existing clinical markers will be essential for clinical translation. Ultimately, combining *MKI67* expression with additional molecular and immune features may enable more precise, biology-driven treatment strategies for ER+/HER2− breast cancer.
